# Characterization of a DsbA family protein reveals its crucial role in oxidative stress tolerance of *Listeria monocytogenes*

**DOI:** 10.1128/spectrum.03060-23

**Published:** 2023-10-12

**Authors:** Jing Xia, Yaru Luo, Mianmian Chen, Yuqing Liu, Zhe Wang, Simin Deng, Jiali Xu, Yue Han, Jing Sun, Lingli Jiang, Houhui Song, Changyong Cheng

**Affiliations:** 1 College of Animal Science and Technology &College of Veterinary Medicine, Zhejiang A&F University, Key Laboratory of Applied Technology on Green-Eco-Healthy Animal Husbandry of Zhejiang Province, Zhejiang Provincial Engineering Research Center for Animal Health Diagnostics & Advanced Technology, Zhejiang International Science and Technology Cooperation Base for Veterinary Medicine and Health Management, China-Australia Joint Laboratory for Animal Health Big Data Analytics, Hangzhou, Zhejiang, China; 2 Ningbo College of Health Sciences, Ningbo, Zhejiang, China; Dominican University New York, Orangeburg, New York, USA

**Keywords:** *Listeria monocytogenes*, DsbA family protein, oxidative stress tolerance, infection

## Abstract

*Listeria monocytogenes* is a facultative, intracellular foodborne pathogen widely distributed in nature and is responsible for severe invasive infection in humans and animals with a mortality rate as high as 30%. *L. monocytogene*s can adapt to various oxidative stresses since it has evolved oxidative modification and regulation mechanisms. The disulfide bond formation protein (Dsb) is required to catalyze the formation of disulfide bonds to promote oxidative folding or modification of proteins involved in bacterial virulence and survival. However, the functions of Lmo1059, a DsbA family protein of *L. monocytogenes*, remain unknown. In this study, we found that Lmo1059 could efficiently catalyze oxidized glutathione (GSSG) reduction, with the residues Cys36 and Cys39 as the key amino acids for its catalytic activity. Moreover, Lmo1059 plays a critical role in oxidative stress tolerance for *L. monocytogenes*, and Cys36 is the crucial amino acid to participate in this process. When Lmo1059 was deleted, the adhesion and invasion of *L. monocytogenes* were reduced while the cell-to-cell spread was increased, suggesting an intricate role of Lmo1059 during bacterial infection. Altogether, these data indicate that the DsbA family protein Lmo1059 is an important participant in the antioxidant stress system and works in concert with other proteins to help protect *L. monocytogenes* from oxidative stress and establish intracellular infection.

**IMPORTANCE:**

The adaption and tolerance to various environmental stresses are the fundamental factors for the widespread existence of *Listeria monocytogenes*. Anti-oxidative stress is the critical mechanism for the survival and pathogenesis of *L. monocytogenes*. The thioredoxin (Trx) and glutaredoxin (Grx) systems are known to contribute to the anti-oxidative stress of *L. monocytogenes*, but whether the Dsb system has similar roles remains unknown. This study demonstrated that the DsbA family protein Lmo1059 of *L. monocytogenes* participates in bacterial oxidative stress tolerance, with Cys36 as the key amino acid of its catalytic activity and anti-oxidative stress ability. It is worth noting that Lmo1059 was involved in the invading and cell-to-cell spread of *L. monocytogenes*. This study lays a foundation for further understanding the specific mechanisms of oxidative cysteine repair and antioxidant stress regulation of *L. monocytogenes*, which contributes to an in-depth understanding of the environmental adaptation mechanisms for foodborne bacterial pathogens.

## INTRODUCTION

*Listeria monocytogenes* (*L. monocytogenes*) is a widespread foodborne intracellular pathogen that can cause severe invasive infections in humans and animals, known as listeriosis (1). *L. monocytogenes* can survive and proliferate in extreme environments such as low pH and temperature, high osmotic pressure, and oxidation. These bacteria can be isolated from various sources including plants, water, soil, and various foods, so it is easy for people and animals to ingest them through food, resulting in infection (2 – 4). When *L. monocytogenes* infects the host, it first passes through the intestinal mucosa, enters the blood circulation, and then proliferates and spreads in target organs such as the liver and spleen. It can cross the blood-brain and placental barriers, resulting in severe symptoms such as sepsis, meningitis, and abortion (5). *L. monocytogenes* mainly infects immunocompromised people, including the elderly, and pregnant women and is one of the foodborne pathogens with the highest mortality (20–30%) (6). Therefore, the research for preventing and controlling this pathogen has fundamental public health significance (7).

*L. monocytogenes* is an important foodborne pathogenic microorganism, and anti-oxidative stress is one of the critical mechanisms of bacterial survival and pathogenicity. In addition to surviving and proliferating in various extreme environments *in vitro*, *L. monocytogenes* can colonize the digestive tract of the hosts and infect macrophages and non-phagocytic epithelial cells (8). When *L. monocytogenes* invades the host digestive tract and phagocytes such as macrophages, host cells can make use of a variety of strategies to overcome bacterial invasions, such as producing reactive oxygen species (ROS), reactive nitrogen species, and reactive chlorine species (RCS) (9). Under oxidative environment, the sulfhydryl (-SH) groups of two cysteine (Cys) residues on the protein peptide chain can be oxidized to form disulfide bonds. Disulfide bonds are essential for protein folding and formation and maintenance of advanced conformation and are switches that regulate specific functions and activities of proteins (10, 11). However, -SH groups of two Cys residues are particularly susceptible to ROS and RCS. Excessive oxidative stress can lead to the covalent modification of cysteine residues, resulting in protein destabilization and inactivation (9). Hence, while disulfide bonds play a crucial role in maintaining protein structure and function, it is important to consider the potential detrimental effects of oxidative damage caused by ROS and RCS on protein integrity.

As a foodborne intracellular parasite, how do *L. monocytogenes* respond to oxidative stress *in vitro* and *in vivo*? Bacteria can avoid excessive oxidation of thiols (containing -SH compounds) by using their sophisticated redox homeostasis repair system. Thiol-disulfide oxidoreductases (TDORs) are the main participants in this repair process (12). The TDORs in bacteria include the thioredoxin (Trx) system, glutaredoxin (Grx) system, and disulfide bond formation protein (Dsb) proteins, which are commonly referred to as the thioredoxin superfamily (13, 14). The thioredoxin superfamily shares a similar fold and contains a -CXXC- active site (15). They are involved in scavenging peroxide-active -SH groups and activating transcription of genes related to anti-oxidative stress to protect damaged proteins from inactivation and prevent aggregation of misfolded proteins (16). These proteins also constitute a complex regulatory network of anti-oxidative stress in *L. monocytogenes*.

Dsb is a protein that can catalyze the formation of disulfide bonds. New peptide chains containing Cys are folded by forming disulfide bonds to form functional proteins with mature conformation, which play a crucial role in the life cycle. The CXXC catalytic motif of Dsb proteins is used to form disulfide bonds in nascent proteins (17, 18). Oxidative folding of proteins to maintain normal protein functions depends on Dsb in most organisms (19). For many bacteria, Dsb plays a folding and stabilizing role in critical cellular processes such as cell division, molecular entry into the cell, response to environmental threats, and assembly of the outer membrane of Gram-negative bacteria. Most bacteria express a wide range of virulence factors containing disulfide bonds including secretory toxins, surface components, such as adhesins and pili, and secretory systems, which are involved in bacterial adhesion, host cell regulation, intercellular spread, and survival (10, 12, 20, 21). In other words, Dsb is required for correct function of other virulence factors and can be regarded as a virulence factor itself.

The Trx and Grx systems have been reported to be involved in the anti-oxidative stress and pathogenicity of *L. monocytogenes*. The former (Trx system) can resist oxidative stress by regulating the protein -SH/disulfide bond balance (22, 23). The latter (Grx system) protects protein -SH groups from irreversible oxidation by catalyzing the reversible exchange of -SH/disulfide bonds between protein -SH groups and abundant reduced (GSH) and oxidized glutathione (GSSG) (24, 25). However, whether the Dsb system is involved in anti-oxidative stress and pathogenicity in *L. monocytogenes* remains unclear. Based on the *L. monocytogenes* EGD-e genome, except for Lmo0964 annotated as the “DsbA-like” protein, only Lmo1059 was annotated as a DsbA family protein. YjbH, encoded by *lmo0964*, was first demonstrated to be necessary for ActA translation (26). Further reports have clarified that YjbH directly interacts with SpxA1 and plays a pivotal role in bacterial adaption to oxidative stress and host infection (27, 28). However, the function of Lmo1059, the DsbA family protein of *L. monocytogenes*, remains unknown. In this study, we investigated the function of Lmo1059, and the data for the first time elucidates that the DsbA family protein Lmo1059 is highly involved in oxidative stress tolerance and helps to establish intracellular infection in *L. monocytogenes*.

## MATERIALS AND METHODS

### Bacterial strains, plasmids, primers, and culture conditions

*L. monocytogenes* EGD-e was used as the wild-type strain. *Escherichia coli* DH5α was employed for cloning experiments and as the host strain for plasmids pET30a(+), pIMK2, and pKSV7. *E. coli* Rosetta (DE3) was used for prokaryotic protein expression. *Listeria* strains were grown in brain heart infusion (BHI) medium (Oxoid, Hampshire, England). *E. coli* strains were cultured at 37℃ in Luria-Bertani broth (LB) (Oxoid). Ampicillin (50 µg/mL), kanamycin (50 µg/mL), or chloramphenicol (10 µg/mL) were added to the media by final concentration when necessary. All chemicals were obtained from Sangon Biotech (Shanghai, China), Merck, or Sigma-Aldrich (St. Louis, MO, USA) and were of the highest available purity. All primers used in this study are listed in Table S1.

### Bioinformatics analysis

The amino acid sequences of thioredoxin from *L. monocytogenes* EGD-e and DsbA family proteins in other microbial species were obtained from the National Centre for Biotechnology Information database. The amino acid sequences and key amino acid sites were aligned with CLC Sequence Viewer software.

### Prokaryotic expression and purification of recombinant proteins

The recombinant proteins used in this study were expressed as an N-terminal His tag fusion using pET30a(+) as the expression vector and Rosetta (DE3) as the expression host. The full-length ORF of the gene of interest from the EGD-e genome was amplified with the primer pair, inserted into the pET30a(+) vector, and finally transformed into Rosetta-competent cells. *E. coli* cells harboring recombinant plasmids were grown in 500 mL LB supplemented with 50 µg/mL kanamycin at 37℃ until cultures reached 0.8–1.0 at OD_600 nm_. Isopropyl β-D-1thiogalactopyranoside was added to a final concentration of 0.2 mM to induce the expression of recombinant proteins for an additional 3 h under optimized conditions. His-tagged fusion proteins were purified using nickel-chelated affinity column chromatography.

### Site-directed mutagenesis

To verify whether Cys36 and Cys39 are the key amino acid sites of Lmo1059, single mutants (C36S and C39S) were generated using the original vector template, pET30a-Lmo1059, and the mutant protein expression vectors were constructed with the primer pairs (Lmo1059-C36S fwd/rev and Lmo1059-C39S fwd/rev) described in Table S1. All mutant constructs were sequenced to ensure that only the desired single mutations had been incorporated correctly after PCR amplification and plasmid transformation were performed. The corresponding mutant proteins were designated Lmo1059_C36S_ and Lmo1059_C39S_, expressed and purified as described above.

### *In vitro* reductase activity assays

The ability of Lmo1059 to catalyze the reduction of human insulin (Sigma) in the presence of dithiothreitol (DTT) was measured as described previously (23). Briefly, reaction mixtures were prepared using 0.1 M potassium phosphate buffer (pH 7.0), 150 µM insulin, 2 mM EDTA, and 1.0 µM purified proteins in a final volume of 200 µL. Reactions were initiated by adding DTT to a final concentration of 1 mM and monitored as the increase in absorbance at 650 nm every 10 min (150 min in total) at 25℃ using the Micro-plate reader Synergy H1 (BioTek Solutions, Inc., Santa Barbara, CA, USA). The TrxA catalyzation and non-enzymatic reduction of insulin by DTT were used as positive and negative controls, respectively.

The ability of Lmo1059 to catalyze the reduction of oxidized glutathione (GSSG) by DTT was also measured. The DTT/glutathione equilibrium lies far on the side of oxidized DTT, and DTTox formation can be followed spectrophotometrically. GSSG (5 mM) was reduced by DTT (5 mM) at 25℃ in 100 mM formic acid/NaOH (pH 4.0), 1 mM EDTA. Reactions were monitored based on the increase in absorbance at 287 nm. The TrxA catalyzation and only DTT were both used as negative controls.

### Construction of gene deletion mutants

Construction of *L. monocytogenes* gene deletion mutants was performed as described previously (27). The temperature-sensitive pKSV7 shuttle vector generated mutations in the *L. monocytogenes* strain EGD-e. A homologous recombination strategy with the splicing by overlap extension (SOE) PCR procedure was used for in-frame deletion to construct gene deletion mutants. The recombinant plasmid containing the target gene deletion cassette was transformed into *E. coli* DH5α. After confirmation by sequencing, the recombinant vector was then electroporated into the competent *L. monocytogenes* EGD-e cells. Transformants were selected on BHI agar plates containing chloramphenicol (10 µg/mL). After serially passaged at 42℃ to promote chromosomal integration, then at 30℃ to enable plasmid excision and curing. The recombinants were identified as chloramphenicol-sensitive colonies, and the mutagenesis was further confirmed by PCR and DNA sequencing.

### Complementation of gene deletion and site-directed mutagenesis mutants

To complement the *L. monocytogenes* Δ*lmo1059* strain, we constructed the complement strains using the integrative plasmid pIMK2. The complete ORF of *lmo1059* and its promoter region were amplified and cloned into pIMK2 following digestion with the appropriate restriction enzymes to remove the P*help* region. The resulting plasmids were electroporated into the *L. monocytogenes* Δ*lmo1059* strain. Regenerated cells were plated on BHI agar containing kanamycin (50 µg/mL). The complemented strain was designated CΔ*lmo1059*. For site-directed mutagenesis complement strains, The C36S or C39S mutation was introduced into the complemented plasmid and then electroporated into the *L. monocytogenes* Δ*lmo1059* strain. The complement strains were verified by sequencing and named as CΔ*lmo1059*_C36S_ and CΔ*lmo1059*_C39S_.

### Bacterial morphology

*L. monocytogenes* wild-type and gene deletion mutant strains were grown on the BHI agar plates for 12 h, and the bacterial colony morphology was observed by using a stereomicroscope. The size of single bacterial colony was measured at 100 bacteria per condition.

### Survival ability of *L. monocytogenes* under oxidative conditions

For oxidative stress, H_2_O_2_ was used as a direct oxidant and diamide as a thiol-specific oxidizing agent. *L. monocytogenes* EGD-e, mutant Δ*lmo1059,* and the complemented strain CΔ*lmo1059* were grown overnight at 37℃ in BHI broth with shaking. Cultures were collected by centrifugation at 5,000 × *g* at 4℃, washed two times in phosphate-buffered saline (PBS) (10 mM, pH 7.4) and the initial OD_600 nm_ was adjusted to 0.6 with PBS. Bacteria were diluted (1:100) in BHI broth containing 10 mM H_2_O_2_, and incubated at 37℃ for 12 h. Kinetic growth at OD_600 nm_ was measured at 1 h intervals. For diamide stress, bacteria were serially diluted 10-fold, and 10 µL of each dilution was spotted onto BHI agar plates containing diamide (1 mM, 1.5 mM, and 2 mM), and incubated at 37℃ for 24 h. Oxidative tolerance of wild-type EGD-e and *lmo1059* cysteine mutants exposed to different concentrations of diamide (0.5 mM, 1 mM, 1.5 mM, and 2 mM) was performed the same as described above. The growth curves in liquid medium are shown with logarithmic *Y*-axis. Also, growth rates in exponential phase of three independent cultures per strain plus statistical analysis for comparison are calculated using GraphPad Prism 8.0. For the apparently restrained growth curves, the growth rate and lag time were simultaneously considered for statistical analysis.

### Proliferation in RAW264.7 macrophages

Stationary phase *L. monocytogenes* were washed and re-suspended in 10 mM PBS (pH 7.4). Monolayers of RAW264.7 cells cultured in Dulbecco’s modified Eagle’s medium (DMEM) (Thermo Fisher Scientific) containing 10% fetal bovine serum (FBS) (HyClone, Chicago, IL, USA). Cells were then infected with bacteria at a multiplicity of infection (MOI) of 1:4 for 30 min, washed two times with warmed PBS prior to replacing media, and gentamicin was added at 50 µg/mL 1-h post-infection to kill extracellular bacteria. At 0.5, 2, 5, or 8 h post-infection, cells were lysed by adding 1 mL ice-cold sterile distilled water, and lysates were diluted 10-fold for enumeration of viable bacteria on BHI agar plates. Each data point represents the average of three wells.

### Adhesion, invasion, and survival in Caco-2 cells

Bacterial survival in human intestinal epithelial Caco-2 cells was assessed as described previously (29). Overnight grown *L. monocytogenes* strains were washed and re-suspended in PBS (10 mM, pH 7.4). Monolayers of Caco-2 cells cultured in RPMI1640 containing 20% FBS were infected with bacteria for 30 min with MOI at 10:1. For adhesion, cells were lysed after being washed three times with PBS. For estimation of invasion, cells were infected with bacteria for 90 min. Then cells were incubated in RPMI1640 containing gentamicin at 50 µg/mL for an additional 90 min to kill extracellular bacteria. Caco-2 cells were lysed by adding 1 mL of ice-cold sterile distilled water. Lysates were 10-fold diluted for the enumeration of viable bacteria on BHI agar plates. Adhesion was expressed as the ratio of recovered colonies to colonies inoculated, while the invasion was calculated as the ratio of colonies recovered after gentamicin treatment to colonies inoculated.

### Plaque-forming assay on mouse fibroblasts L929 cells

The plaque assay was performed as previously described (30). Briefly, murine L929 fibroblast cell monolayers were maintained in high-glucose DMEM containing FBS (HyClone) and 2 mM L-glutamine. Cells were plated at 1 × 10^6^ cells per well in a six-well dish and infected at an MOI of 1:2.5 with *L. monocytogenes* under 37°C with 5% CO_2_ for 1 h. Extracellular bacteria were killed with 50 µg/mL gentamicin, and cells were washed two times with warmed PBS (10 mM, pH 7.4) and then overlaid with 3 mL of medium plus 0.7% agarose and 10 µg/mL gentamicin. Cells were fixed with paraformaldehyde (4% in PBS) for 20 min and stained with crystal violet after 48 h of infection for plaque observation. The plaque size and numbers of the mutant strains were indicated as a percentage of those formed by the wild-type strain. The plaque size of wild-type strain EGD-e was set as 100%. Data are expressed as mean ± SD of randomly selected plaques (100 plaques for size comparison) for each strain.

### Virulence in the mouse model

The *L. monocytogenes* strains EGD-e, Δ*lmo1059*, and CΔ*lmo1059* were tested for recovery in liver and spleen sections of ICR mice (female, 18–22 g, purchased from Zhejiang Academy of Medical Sciences, Hangzhou, China). ICR mice (eight per group) were injected intraperitoneally with ∼10^6^ CFU of each strain. For bacterial CFU recovery from organs, mice were sacrificed at 24 and 48 h post-infection, and livers and spleens were removed and individually homogenized in PBS (10 mM, pH 7.4). Surviving bacteria were enumerated by plating serial dilutions of homogenates on BHI agar plates. Results were expressed as mean ± SD of each group’s recovery rate per organ.

### Statistical analysis

All experiments were repeated three times. Data were analyzed using the unpaired two-tailed Student’s *t* test. Differences with *P* < 0.05 were considered statistically significant.

## RESULTS

### Lmo1059 exhibited strong GSSG reductase activity

**Fig 1:**
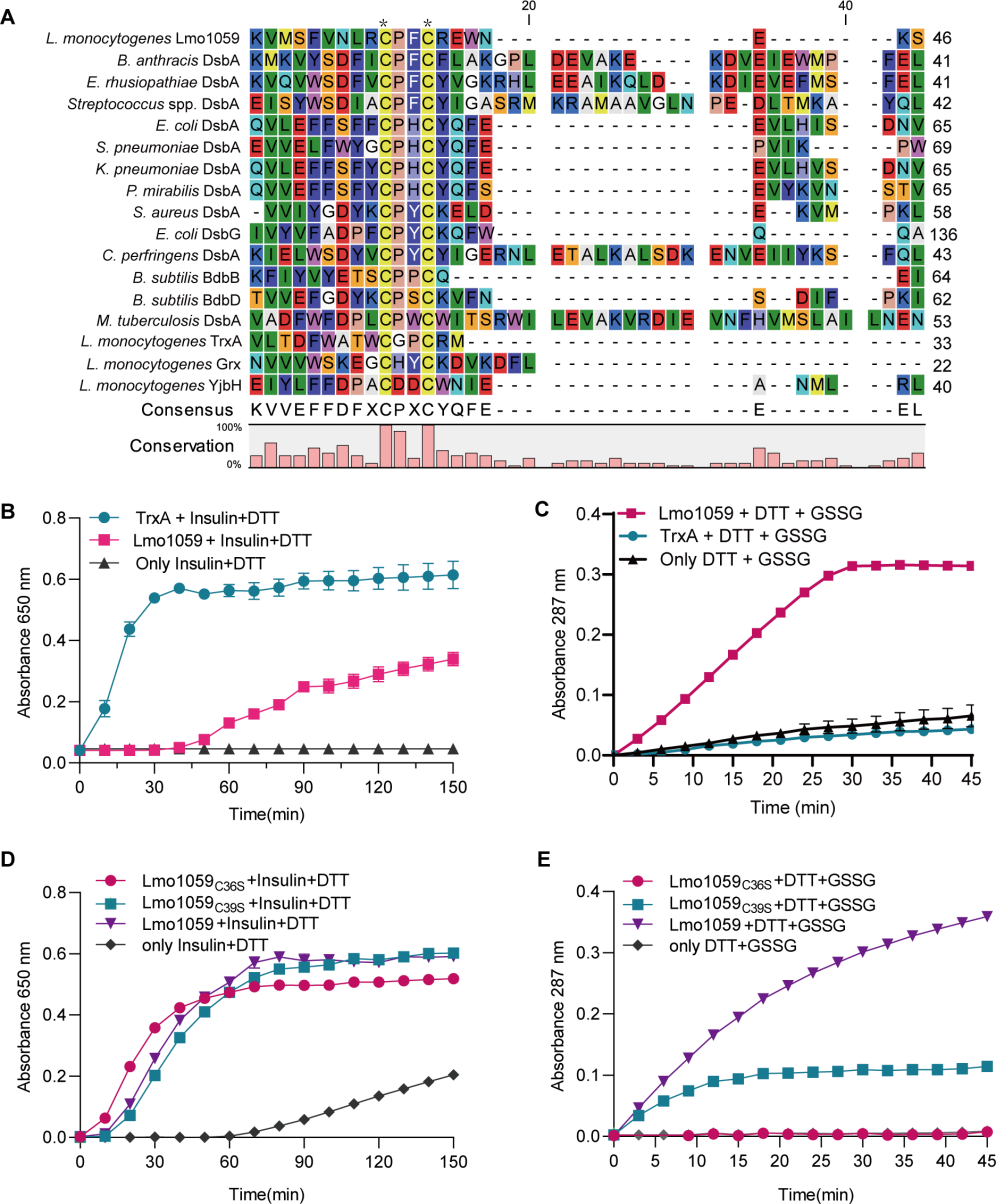
Lmo1059 contains the family conserved CXXC active motifs and exhibits strong GSSG reductase activity. (**A**) Amino acid sequence alignment of *L. monocytogenes* putative DsbA family protein Lmo1059 against homologs from other bacterial species. The conserved CXXC catalytic motifs are denoted with asterisks. *In vitro* insulin disulfide reductase activity (**B and D**) and GSSG reductase activity (**C and E**) of recombinant Lmo1059 and its site-directed mutants (Lmo1059_C36S_ and Lmo1059_C39S_) are shown. The Michaelis-Menten plot and kinetic parameters, Km, Vmax, and Kcat, for Lmo1059 are shown in Fig. S1B and C. *L. monocytogenes* thioredoxin A, TrxA, which has efficient insulin disulfide reductase activity but no GSSG reductase activity, was taken as a reference control in this assay. Data are expressed as mean ± SD for three replicates. Panels (B) and (C) are republished from *Frontiers in Cellular and Infection Microbiology* (23). The two plots were retained to provide a comprehensive display of the related results of this study. The data presented in the current manuscript stem from entirely new experiments, separate from those featured in the previous publication.

As shown in Fig. 1A, Lmo1059 has the typical thioredoxin active motif “CXXC” (CPFC) at positions 36–39 and closely relates to the DsbA homologs of other bacteria species (Fig. S1A). The catalytic data showed that Lmo1059 exhibits the ability to catalyze the reduction of insulin, albeit with low efficiency, as depicted in Fig. 1B. In contrast, Lmo1059 demonstrated vigorous reduction activity of GSSG, as shown in Fig. 1C. The enzyme activity-time curve of Lmo1059 protein showed that the concentration of oxidized DTT in the reaction product increases with the extension of the total reaction time. The Michaelis-Menten for Lmo1059 was plotted, and the kinetic parameters, Km, Vmax, and Kcat, were calculated, which were presented in Fig. S1B and C. The ability of Lmo1059 to reduce GSSG and insulin was previously published (23); data are presented here for keeping a complete display of the related results.

### The Cys36 and Cys39 are the critical amino acids of the GSSG reductase activity of Lmo1059

The Cys36 and Cys39 of Lmo1059 were predicted as the critical active sites through sequence alignment. After site-directed mutagenesis, we found that in the insulin reduction test (Fig. 1D), the enzyme activity curves of the two mutant proteins Lmo1059_C36S_ and Lmo1059_C39S_ showed the same trends to Lmo1059, indicating these specific amino acids are not required for catalyzing the reduction of insulin, albeit with reduced efficiency. However, for the reduction activity of GSSG (Fig. 1E), the enzyme activity curve of mutant Lmo1059_C39S_ was significantly impaired, with only about half the activity of Lmo1059. In contrast, the activity of mutant Lmo1059_C36S_ was completely abolished. These data implied that Cys36 and Cys39, especially Cys36, were the key amino acids of Lmo1059 to catalyze the reduction of GSSG.

### Lmo1059 is not required for *in vitro* growth and colony morphology of *L. monocytogenes*

**Fig 2:**
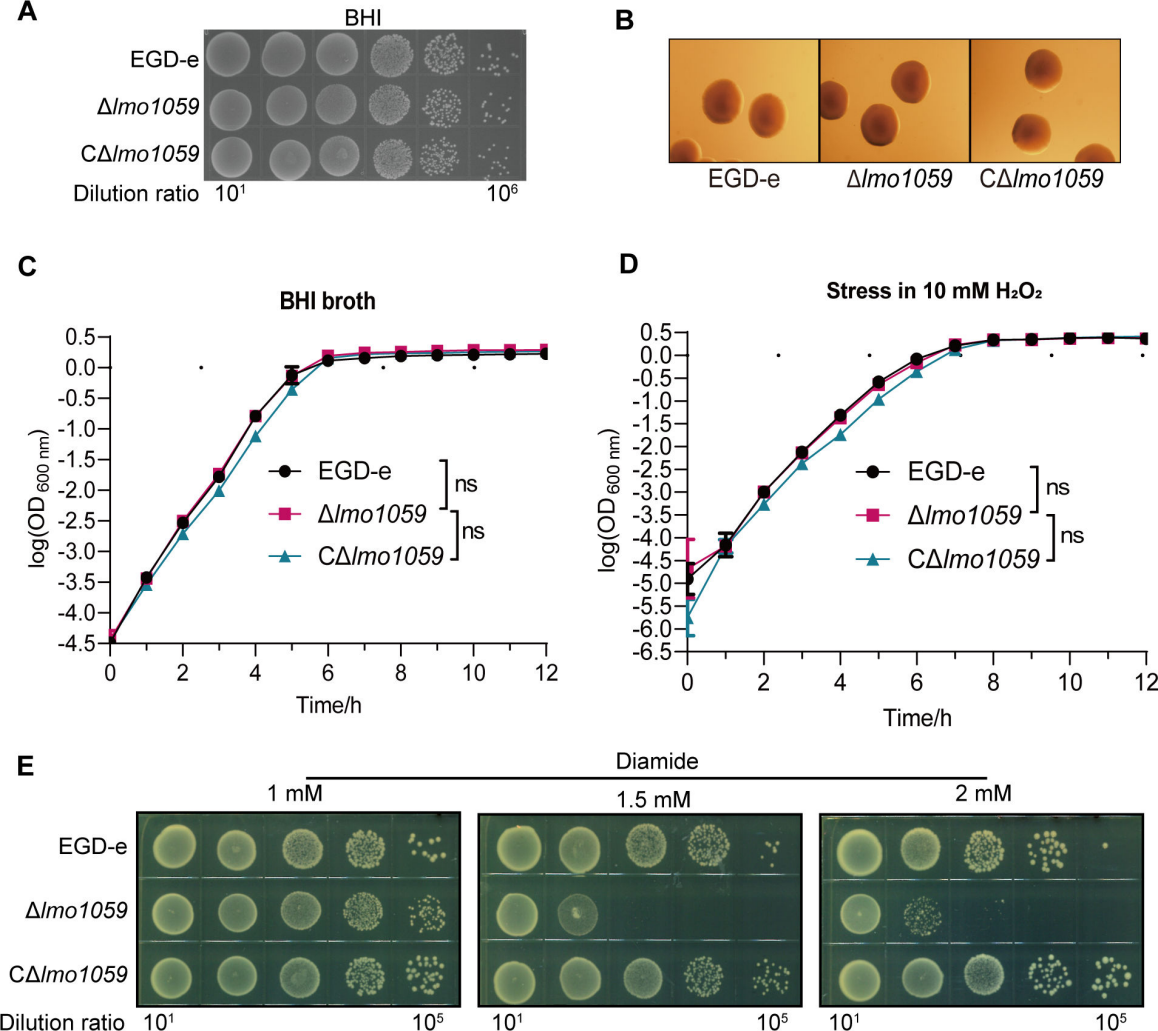
Deletion of *lmo1059* did not affect bacterial *in vitro* growth and colony morphology but decreased the tolerance to oxidative stress for *L. monocytogenes*. (**A, C**) Plate culture and growth curve of wild-type EGD-e and *lmo1059* mutants. (**B**) The bacterial colony morphology grown on the BHI agar plates for 12 h was observed by using a stereomicroscope. (**D, E**) Overnight-grown bacteria were subjected to H_2_O_2_ (**D**) and diamide (**E**) by diluting (1:100) in BHI containing 10 mM H_2_O_2_ or spotting 10-fold dilution CFU of each bacterial strain onto BHI plates containing various concentrations of diamide. (**C, D**) The growth curves are shown with logarithmic *Y*-axis. Data are expressed as mean ± SD for three replicates. Growth rates in exponential phase of three independent cultures per strain plus statistical analysis for comparison are calculated using GraphPad Prism 8.0. ns, no significance. (**A, B, and E**) Representative images of three independent experiments are shown.

The growth curves of Δ*lmo1059* and CΔ*lmo1059* were comparable to the wild-type strain EGD-e, as shown in Fig. 2C. Results of plate culture after 6 h growth in liquid medium showed that *in vitro* growth capacity of *L. monocytogenes* was not affected without *lmo1059* (Fig. 2A), indicating that the deletion of *lmo1059* did not have an impact on the growth of *L. monocytogenes*. To further explore whether the loss of *lmo1059* was involved in the colony morphology, we enlarged the colony under a stereomicroscope and observed that the colony morphology of *lmo1059* mutants was the same as that of EGD-e, with round colonies with smooth edges and similar sizes. The results suggested that the *lmo1059* was not required for the colony morphology of *L. monocytogenes in vitro* (Fig. 2B).

### Lmo1059 is responsible for bacterial resistance to thiol-specific oxidative stress

The survival ability of EGD-e, Δ*lmo1059*, and CΔ*lmo1059* in the medium containing 10 mM H_2_O_2_ were shown in Fig. 2D. There was no difference in the survival of the three strains. The survival capacity under Cu^2+^ and Cd^2+^ stress (used as the redox-active stress) were previously published (23), bacteria in the absence of *lmo1059* remain cadmium- and copper-resistant. The most significant survival difference was found in the thiol-specific oxidizing agent diamide stress. Under 1.5 mM diamide stress, the anti-oxidative capacity of Δ*lmo1059* decreased by three orders of magnitude compared with EGD-e, and the situation was similar under 2 mM diamide stress (Fig. 2E). We observed overcompensation by complementation for some of the phenotypes, specifically under the diamide stress conditions. One possible reason for the observed overcompensation is that the CΔ*lmo1059* strain used in our study was generated using an integrative plasmid. It is worth noting that under oxidative stress, the transcriptional level of the *lmo1059* gene increased compared to the wild-type strain (data not shown). This elevated transcriptional level could potentially lead to higher expression levels of Lmo1059 in the complemented strain, thereby resulting in the observed overcompensation. Collectively, these results revealed that *lmo1059* was involved in bacterial oxidative stress tolerance, specifically for thiol-specific oxidative stress.

### Cys36 is the crucial amino acid for Lmo1059-mediated oxidative stress tolerance in *L. monocytogenes*

**Fig 3:**
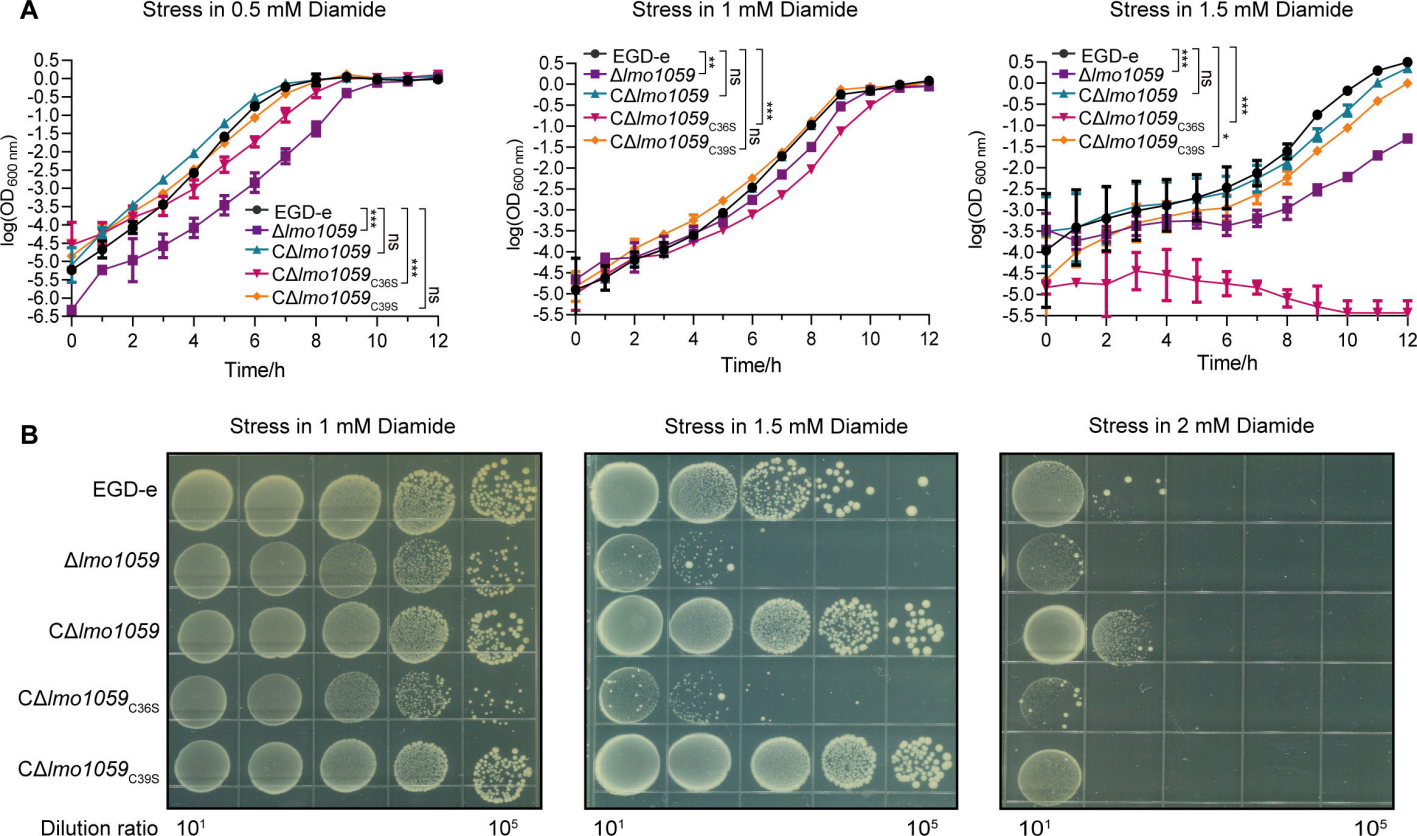
Cys36 is the crucial amino acid for Lmo1059 mediating oxidative stress tolerance in *L. monocytogenes*. Oxidative tolerance of wild-type EGD-e and Lmo1059 cysteine mutants exposed to different concentrations of diamide in liquid BHI medium (**A**) and BHI plates (**B**) are presented. The anti-oxidative stress ability of the cysteine mutant CΔ*lmo1059*_C36S_ was similar to Δ*lmo1059*, significantly lower than that of the wild-type EGD-e and the complemented strain CΔ*lmo1059*. (**A**) Growth curves with logarithmic *Y*-axis are shown. Data are expressed as mean ± SD for three replicates. Also, growth rates in exponential phase of three independent cultures per strain plus statistical analysis for comparison are calculated using GraphPad Prism 8.0. For the apparently restrained growth curves, the growth rate and lag time were simultaneously considered for statistical analysis. ns, no significance, *P < 0.05, **P < 0.01, ***P < 0.001. (**B**) Representative images of three independent experiments are shown.

In the previous results, we found that the two cysteines of Lmo1059 are the key amino acid sites for Lmo1059 to exert its reductase activity, so we speculated that *L. monocytogenes* depended on the two cysteines of Lmo1059 to induce resistance to oxidative stress. We generated the strains CΔ*lmo1059*_C36S_ and CΔ*lmo1059*_C39S_, which are derived from the *lmo1059* deletion mutant and are complemented with the two mutated *lmo1059* variants C36S and C39S. The survival ability was measured in a BHI liquid medium containing 0.5 mM, 1 mM, or 1.5 mM diamide and in a BHI plate containing 1 mM, 1.5 mM, or 2 mM diamide. Survival results under different concentrations of diamide stress are shown in Fig. 3. In the BHI liquid medium containing 0.5 mM and 1 mM diamide, the survival ability of CΔ*lmo1059*_C36S_ and Δ*lmo1059* were significantly slower than those of EGD-e and CΔ*lmo1059*. This phenomenon was more evident under 1.5 mM diamide stress condition, the growth of CΔ*lmo1059*_C36S_ and Δ*lmo1059* strains almost stopped, and CΔ*lmo1059*_C39S_ growth was also impaired (Fig. 3A). On BHI plates with 1.5 mM or 2 mM diamide, the anti-oxidative stress ability of the cysteine mutant CΔ*lmo1059*_C36S_ was similar to Δ*lmo1059*, significantly lower than that of the wild-type strain and the complemented strain CΔ*lmo1059* (Fig. 3B). Therefore, we conclude that Cys36 is the crucial amino acid for Lmo1059-mediated oxidative stress tolerance in *L. monocytogenes*.

### Impact of Lmo1059 on virulence of *L. monocytogenes in vitro* and *in vivo*

**Fig 4:**
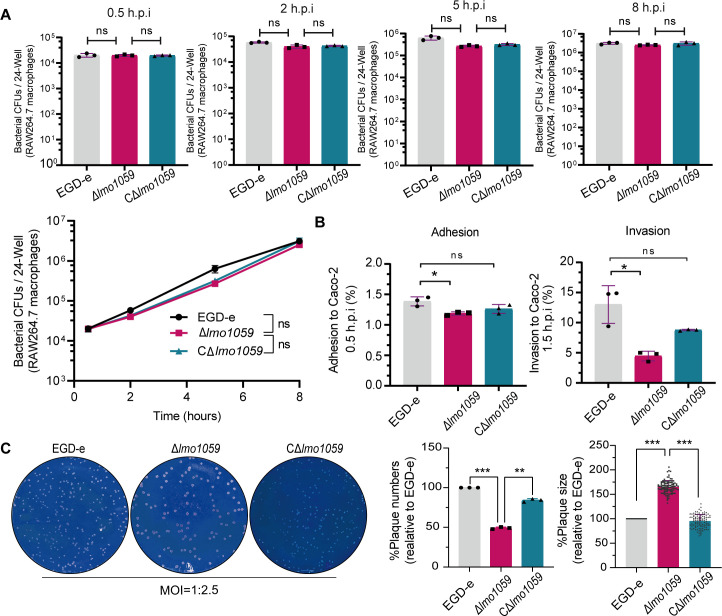
Deletion of Lmo1059 reduced the adhesion and invasion ability of bacteria to Caco-2 cells but significantly increased the efficiency of cell-to-cell spread. (**A**) Intracellular growth of wild-type *L. monocytogenes* and *lmo1059* mutants in RAW264.7 macrophages. The infected macrophages were lysed at 0.5, 2, 5, and 8 hr, and viable bacteria were serially plated on BHI plates. The number of recovered bacteria able to invade cells and survive are expressed as mean ± SD of three replicates for each strain. ns, no significance. (**B**) Adhesion and invasion of *L. monocytogenes* in human intestinal epithelial cells, Caco-2. Cells infected with *L. monocytogenes* strains were lysed at the indicated time points, and viable bacteria were serially plated on BHI agar plates. The number of bacteria able to invade cells and survive are expressed as mean ± SD of the recovery rate for each strain (three replicates). ns, no significance, *P < 0.05. (**C**) The plaque assay was performed using L929 fibroblasts. The plaque size and numbers of the mutant strains were indicated as a percentage of those formed by the wild-type strain. Data are expressed as mean ± SD of randomly-selected plaques (100 plaques for size comparison) for each strain. **P < 0.01, ***P < 0.001. Representative plague formation images of three independent experiments are shown.

Intracellular growth was performed accordingly on RAW264.7 macrophages. The results showed no significant difference in the number of bacteria among EGD-e, Δ*lmo1059*, and CΔ*lmo1059* during infection with *L. monocytogenes* for 0.5, 2, 5, and 8 hr (Fig. 4A), suggesting that deletion of *lmo1059* did not affect the survival and proliferation of *L. monocytogenes* in RAW264.7 cells. Notably, the adhesion and invasion rates of Δ*lmo1059* to Caco-2 cells were significantly lower than the wild-type EGD-e (Fig. 4B), indicating that *lmo1059* contributes to the adhesion and invasion of *L. monocytogenes*.

**Fig 5:**
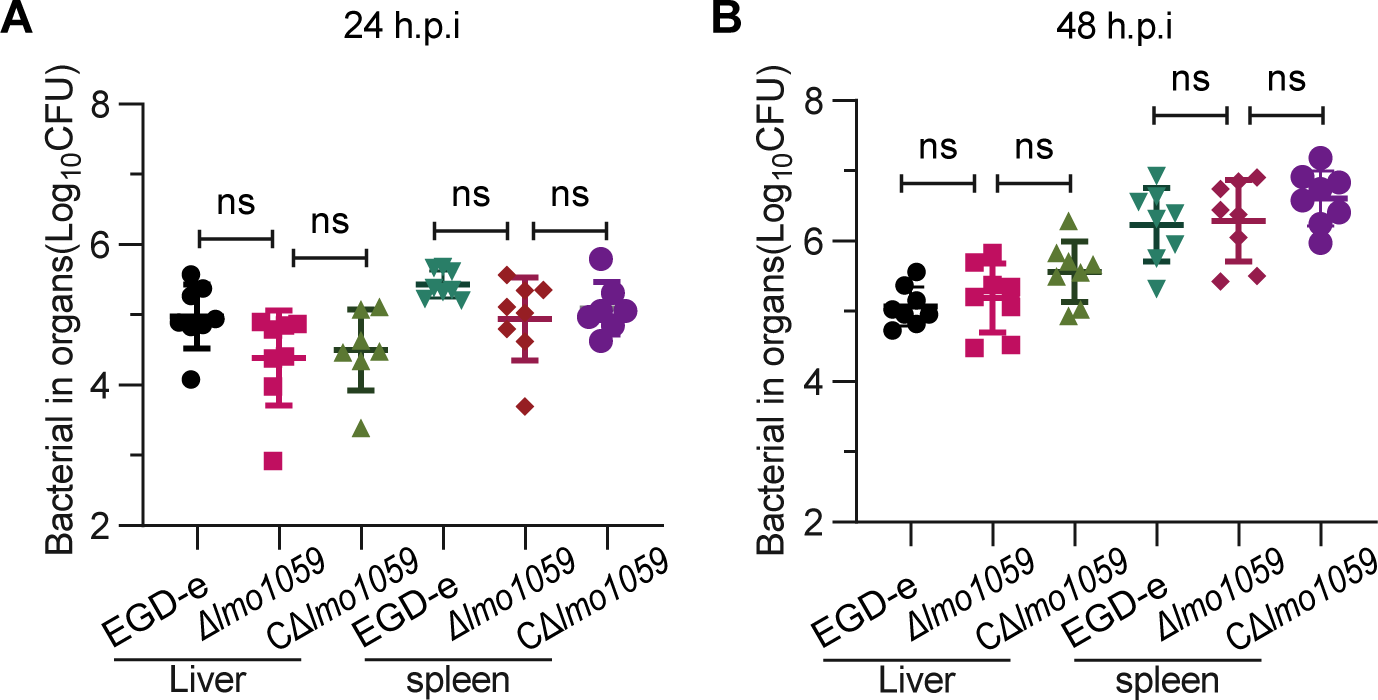
Lmo1059 was not required for bacterial virulence in mice. The wild-type and mutant strains were inoculated intraperitoneally into ICR mice at ~1 × 10^6^ CFU. Animals were euthanized at 24 (**A**) or 48 h (**B**) post-infection, and organs (livers and spleens) were recovered and homogenized. Homogenates were serially diluted and plated on BHI agar. The numbers of bacteria colonizing the organs are expressed as mean ± SD of the log_10_ CFU per organ for each group (eight mice). ns, no significance.

To further assess whether *lmo1059* played a part in cell-to-cell spread efficiency, we tested the ability of *lmo1059* mutant and wild-type strain EGD-e to form plaques on L929 fibroblast monolayers. As indicated in Fig. 4C, the number of plaques produced by Δ*lmo1059* was significantly lower than its parent and complement strains, while the plaque sizes produced by Δ*lmo1059* were significantly increased. The size of the plaques is used as a measure of the efficiency of cell-to-cell spread (31), which suggesting that the lack of *lmo1059* significantly enhanced the ability of bacteria to spread cell-to-cell. Based on the results from the cell experiments, we further explore the virulence of this mutant strain in mice. However, the number of colony-forming units (CFUs) recovered from the spleens and livers of infected mice at 24 and 48 h of infection exhibited no significant difference for the Δ*lmo1059* compared with the wild-type strain (Fig. 5A and B). These data revealed that *lmo1059* participated in bacterial adhesion, invasion, and cell-to-cell spread while contributing no significant role in virulence in mice overall.

## DISCUSSION

According to the previous report, through sequence analysis and structure prediction, 14 proteins with CXXC active motifs in *L. monocytogenes* EGD-e were found to be involved in redox reaction, most of which were predicted to be Trx or Grx (32). Among them, the thiol:disulfide oxidoreductase-like proteins Lmo0964 and Lmo1059 are predicted to be DsbA family proteins (32). Lmo0964, namely YjbH, was first demonstrated to be necessary for ActA translation (26). Further reports have clarified that YjbH directly interacts with SpxA1 and plays a pivotal role in bacterial adaption to oxidative stress and host infection (27, 28). Here, we aimed to investigate the biological roles of Lmo1059 in oxidative stress tolerance and infection.

The choice to test both insulin and GSSG as substrates for Lmo1059 was based on the understanding that DsbA family proteins are involved in the reduction of disulfide bonds in proteins and the maintenance of cellular redox balance. Insulin reduction assay is a widely used method to assess the reduction activity of proteins, and it helps to determine whether Lmo1059 possesses general reductase activity (33). Our results showed that Lmo1059 has the ability to reduce insulin, albeit with low efficiency (Fig. 1B). This finding suggests that Lmo1059 may have some reductase activity towards protein disulfide bonds, but it may not be the main substrate of Lmo1059 *in vivo*. To explore the potential functions of Lmo1059 in cellular redox processes, we also investigated its activity in reducing GSSG, which is a key oxidant involved in cellular redox homeostasis (34). The strong GSSG reductase activity of Lmo1059 (Fig. 1C) indicates that Lmo1059 is capable of participating in the reduction of GSSG and potentially plays a role in maintaining cellular redox balance. In contrast to TrxA and Grx in *L. monocytogenes*, Lmo1059 exhibits a strong reduction activity towards GSSG (23, 25). Typically, the active site with two cysteines separated by two amino acids (CXXC) is crucial for TDORs (12). In the case of TrxA in *L. monocytogenes*, both C28A and C31A mutations completely abolish its oxidoreductase ability to catalyze the reduction of insulin (23). In the case of Lmo1059, it has been observed that the two cysteines (Cys36 and Cys39, particularly Cys36) are required for its catalytic activity against GSSG but not insulin. This suggests that Lmo1059 differs from other oxidoreductase-like proteins in *L. monocytogenes*, exhibiting distinct enzymatic characteristics.

DsbA family proteins have important roles in maintaining bacteria redox homeostasis under oxidative stress and are required for different types of oxidative stress in bacteria. *Mycobacterium tuberculosis rv2466c* gene, encoding an oxidoreductase enzyme annotated as DsbA, is essential for bacterial survival under H_2_O_2_ stress (35). In *Neisseria meningitidis*, the *dsbA1A2* mutant was more sensitive to paraquat and copper but not to hydrogen peroxide relative to the wild-type strain (36), while FrnE (putative DsbA protein) deletion in *D. radiodurans* was sensitive to Cd^2+^ and other oxidative stress producing agents, like diamide, gamma rays, and hydrogen peroxide, albeit to different levels (37). Meanwhile, a putative DsbA (encoded by *ncgl0018*) deleted strain exhibited a significant increase in sensitivity to all tested chemical reagents (H_2_O_2_, HClO, diamide, CdCl_2_, etc.) as compared with WT strain in *Corynebacterium glutamicum* (38). In this study, our results showed that Lmo1059 was correlated to the tolerance of thiol-specific oxidizing agent diamide, as the Δ*lmo1059* mutant significantly decreased the tolerance to 1.5 and 2 mM diamide stress, similar to TrxA (23), while previous studies in our laboratory have proved that YjbH plays a vital role in tolerance to Cd^2+^ and Cu^2+^ stress (27). Unexpectedly, the deletion of Grx remarkably increased tolerance to various oxidizing agents, including diamide, copper, and cadmium ions, but not to hydrogen peroxide (25). These indicate that different TDORs in *L. monocytogene*s play synergistic roles in helping the bacteria resist different oxidative stress in the environments.

The Cys36 and Cys39 are the key sites of Lmo1059 reductase activity, and the Cys36 of Lmo1059 was crucial for resistance to diamide oxidative stress. Diamide is an electrophilic compound that reacts with protein thiolates by forming disulfide bonds and consequently depletes cellular protein thiolates. Two cysteine residues of *Bacillus subtilis* YodB are involved in oxidative stress response when exposed to diamide stress. Cys6 residues at the N-terminus of the helix could activate the nucleophile of the -SH of Cys and directly sense the sulfhydryl reactive electrophiles (39). Under diamide stress, the N-terminal Cys mutation of Lmo1059 strongly impaired the antioxidant stress ability of *L. monocytogenes*, suggesting that Lmo1059 Cys36 was similar to that of *B. subtilis* YodB Cys6. Based on these, we speculated that the Cys36 mutation of Lmo1059 weakened the formation of intermolecular disulfide bonds with oxidized proteins.

The majority of studies on Dsb proteins and virulence have focused on Gram-negative bacteria, particularly those with Dsb proteins located in the periplasmic space. In Gram-negative bacteria, the periplasmic Dsb proteins play a critical role in the folding and disulfide bond formation of secreted proteins involved in virulence. These Dsb proteins facilitate the correct folding and stability of virulence factors such as adhesins, toxins, and invasins, contributing to the pathogenicity of these bacteria. Deletion of DsbA exhibited attenuated virulence in *E. coli* (40), *Klebsiella pneumoniae* (41), *Salmonella enterica* (42), *Francisella tularensis* (42), *C. jejuni* (43), and many more (10, 11). On the other hand, there is limited information available regarding the role of cytoplasmic Dsb proteins in the virulence of Gram-positive bacteria. The cellular architecture of Gram-positive bacteria, with a single membrane and lack of a periplasmic space, poses distinct challenges and differences in the redox systems involved in protein folding and disulfide bond formation (12). The CcdA proteins (Dsb-like protein) contribute to cytochrome c maturation, virulence regulation and sporulation in pathogenic *Bacillus* spp. strains (44). In *Corynebacterium diphtheriae*, deletion of MdbA (Dsb-like protein) results in a severe temperature-sensitive cell division phenotype. The mutant also fails to assemble pilus structures, thus is greatly defective in toxin production and attenuated in a guinea pig model of diphtheritic toxemia (45). Therefore, it would be valuable to further investigate and elucidate the specific roles and mechanisms of cytoplasmic Dsb proteins in Gram-positive bacteria, including their involvement in virulence. These studies could provide a better understanding of the unique redox systems in Gram-positive pathogens and their contributions to pathogenesis.

*L. monocytogenes* tend to regulate different genes to enhance their survival, especially in harsh environments, since the virulence genes can be upregulated to promote survival (46 – 48). The internalization of *L. monocytogenes* into non-phagocytes is mediated by the internalins, InlA, and InlB, and their cell surface receptors (49). InlB alone can induce the endocytosis of *L. monocytogenes*, while InlA can promote the internalization of *L. monocytogenes* into host cells in the early stage and requires the assistance of other internalin proteins (such as InlC, InlG, and InlH) (50). SigB and PrfA also participate in the invasion of *L. monocytogenes* by controlling the expression of InlA and InlB (51). This study showed that the adhesion and invasion of *L. monocytogenes* were significantly weakened when *lmo1059* was deleted, indicating that *lmo1059* could be involved in the regulation of expression of InlA and InlB by establishing the regulatory network with SigB and PrfA. The intracellular spread of *L. monocytogenes* is dominated by ActA and InlC. The former can form a “comet tail” to provide power for its movement between cells, while the latter can damage the host cytoskeleton to help *L. monocytogenes* enter neighboring cells (52, 53), which depends on the structurally intact eukaryotic cells for the proper formation of *L monocytogenes* actin-rich membrane protrusions (31, 54). Surprisingly, we found that the cell-to-cell spread of *L. monocytogenes* was enhanced without *lmo1059*, suggesting that *lmo1059* could be underlying the regulation of ActA and InlC or indirectly affecting structural integrity within the *Listeria* membrane protrusions.

By analyzing the protein sequences, we found that InlA does not contain any cysteine residues, while InlB contains four cysteine residues. Other internalin proteins, such as InlC, InlI, and InlJ, possess 4, 8, and 14 cysteine residues, respectively. In terms of the regulatory proteins, the virulence central regulator PrfA contains four cysteine residues (55). Previous studies have demonstrated the critical role of TrxA in reducing the intermolecular disulfide bonds of PrfA, leading to the maintenance of its intracellular monomeric state for subsequent dimerization (23). Considering the results obtained from the adhesion, invasion, and cell-to-cell spread assays, we speculate that ActA, InlB, InlC, or other internalin proteins such as InlJ and InlI could potentially be the virulence factors targeted by Lmo1059. However, it is important to note that our current results have not provided conclusive evidence to support these hypotheses. Further experiments are needed to investigate and validate these potential interactions. Meanwhile, our findings indicate that while Lmo1059 affects certain virulence features such as adhesion to and invasion of non-phagocytic cells, as well as cell-to-cell spread, there is no significant impact on other aspects such as survival in macrophages and *in vivo* virulence in mice. This suggests that Lmo1059 may primarily function in oxidizing proteins to ensure their proper functionality, rather than acting as a direct “virulence factor” itself in *L. monocytogenes*. The lack of significant differences in the overall virulence of *Listeria* strains with and without Lmo1059 further supports this notion. Instead, our results suggest that Lmo1059 plays a more prominent role in conferring resistance to oxidative stress in the environment, emphasizing its importance in combating antioxidant stress.

In this study, we first demonstrated that Lmo1059 has vigorous reductase activity to GSSG, which plays a crucial role in helping bacteria resist oxidative stress. The Cys36 of Lmo1059 is the key amino acid for the enzyme activity and resistance to the diamide stress of *L. monocytogenes*. Although the bacterial adhesion and invasion ability were reduced, and the cell-to-cell spread was significantly enhanced, there was no significant difference in organ proliferation after the deletion of Lmo1059. More importantly, Lmo1059 could work with other thioredoxin family members to help protect the bacteria from oxidative stress, which provides novel insights for better understanding the environmental adaptation mechanisms of *L. monocytogenes*.

## SUPPLEMENTAL MATERIAL

Supplemental material spectrum.03060-23-s0001.docx Table S1 and Fig. S1.

ASM does not own the copyrights to Supplemental Material that may be linked to, or accessed through, an article. The authors have granted ASM a non-exclusive, world-wide license to publish the Supplemental Material files. Please contact the corresponding author directly for reuse.
