# Supplementary material for: Characterization of a DsbA family protein reveals its crucial role in oxidative stress tolerance of Listeria monocytogenes
Source: Microbiol Spectr. 2023 Oct 12;11(6):e03060-23. doi: 10.1128/spectrum.03060-23 (PMC10715225; doi:10.1128/spectrum.03060-23)
Supplement: Supplemental material — Table S1 and Fig. S1. [file spectrum.03060-23-s0001.docx]

**Table S1**. Primers used in this study

| Primers | Sequences(5′→3′) |
| --- | --- |
| Lmo1059-fwd | TTTCATATGGATATTAGTCAAATTAAAGCAGAAG |
| Lmo1059-rev | CCGCTCGAGTTTAGCTAATTCATCATCAAGTAGCG |
| Lmo1059-C36S-fwd | ACTTACGTaGCCCATTCTGTCGTGAGTGGAAT |
| Lmo1059-C36S-rev | GAATGGGCtACGTAAGTTAACGAAAGACATTACTTTAAC |
| Lmo1059-C39S-fwd | CCCATTCTcTCGTGAGTGGAATGAAAAATCCA |
| Lmo1059-C39S-rev | ACTCACGAgAGAATGGGCAACGTAAGTTAACG |
| Δ*lmo1059*-up-*Bam*HⅠ-fwd | TTGGGATCCAAGTGAAAGAAGCATACGGTTTAGA |
| Δ*lmo1059*-up-rev | CATCATCAAGTAGCGAACGTAACTCTACTTCTGCTTTAATTTGACTAATATCCA |
| Δ*lmo1059*-down-fwd | TTAAAGCAGAAGTAGAGTTACGTTCGCTACTTGATGATGAATTAGCTAAATA |
| Δ*lmo1059*-*Hin*dⅢ-down-rev | TTCAAGCTTTCATCTTCTTCAGAGAGCGCCGTCA |
| Δ*lmo1059*-a-front | ATCAAATTATTACGCTTGGCACCATG |
| M13-fwd | GTTTCCCAGTCACGAC |
| M13-rev | AGCGGATAACAATTTTCACACAGGA |
| C∆*lmo1059*_P_native_-up-*Sac*I-fwd | GCGAGCTCAGTTATGTACTACCAATCTTCTTCTGTTTT |
| C∆*lmo1059*_P_native_-up-rev | TGACTAATATCCATATGCGTCTCCTCCTCATCAA |
| C∆*lmo1059*_P_native_-down-fwd | GGAGGAGACGCATATGGATATTAGTCAAATTAAAGCAGAAGTAGT |
| C∆*lmo1059*_P_native_-down-*Bam*HI-rev | CGGGATCCTTATTTAGCTAATTCATCATCAAGTAGCGAACG |


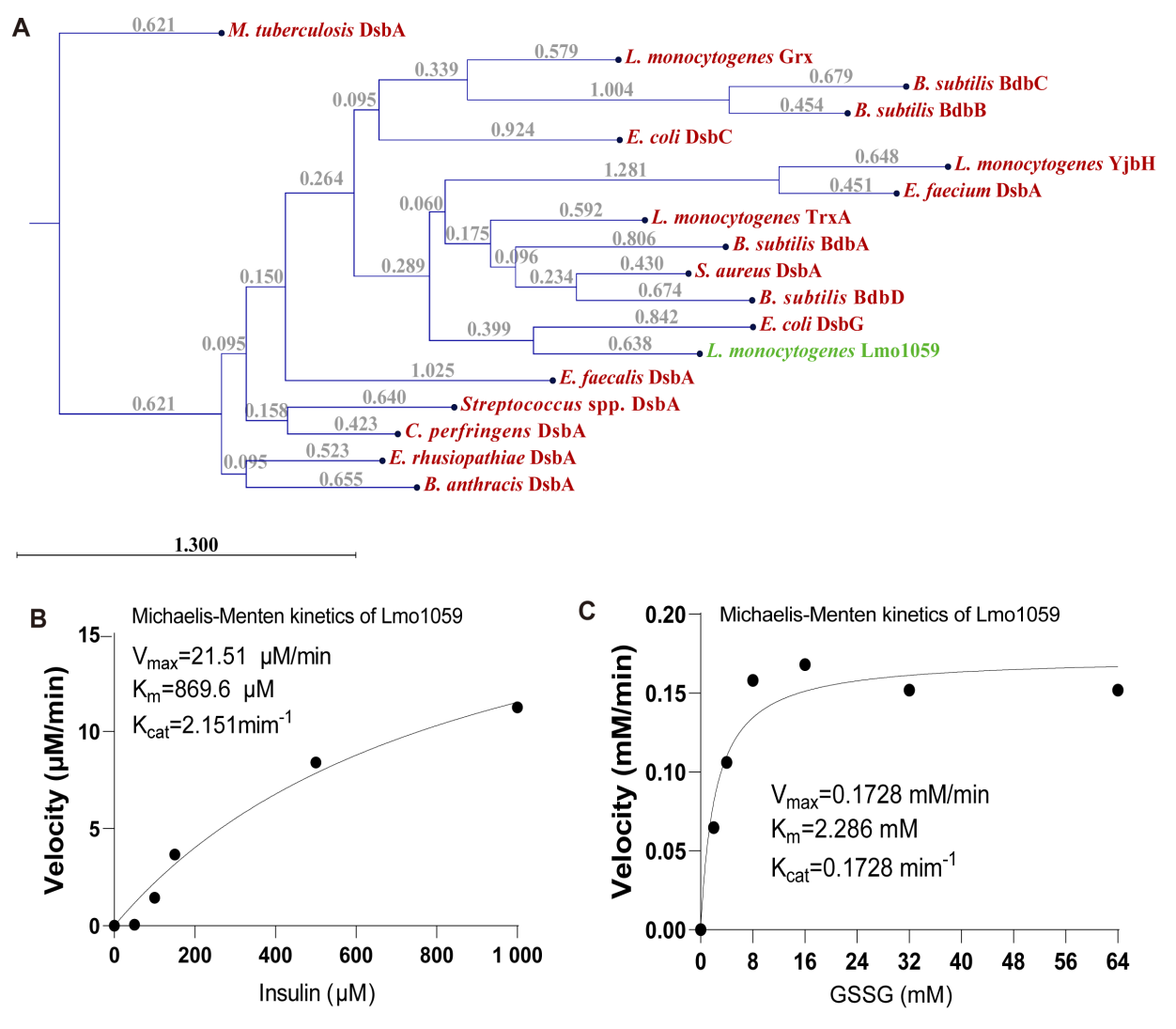


**Figure S1**. **Phylogenetic analysis of DsbA homologs and the fitted Michaelis-Menten plots of Lmo1059.** (A) The phylogenetic tree of the amino acid sequences of thioredoxin from *L. monocytogenes* EGD-e and DsbA family proteins from the above bacterial species. The tree was constructed with the Neighbor-Joining (NJ) program, and a bootstrap test of 100 replicates was used to estimate the confidence of branching patterns, where the numbers on internal nodes represent the support values. (B-C) The Michaelis-Menten plot and kinetic parameters (Km, Vmax, and Kcat) of Lmo1059 in catalyzing the reduction of insulin and GSSG are shown, respectively.
